# Materiality matters: Blurred boundaries and the domestication of functional foods

**DOI:** 10.1057/biosoc.2015.7

**Published:** 2015-04-20

**Authors:** Kate Weiner, Catherine Will

**Affiliations:** a Department of Sociological Studies, University of Sheffield, Elmfield, Northumberland Road, Sheffield S10 2TU, UK; b Department of Sociology, Freeman Building, University of Sussex, Brighton, BN1 9QE, UK. E-mail: c.will@sussex.ac.uk

**Keywords:** cholesterol, functional food, materiality, script, practices

## Abstract

Previous scholarship on novel foods, including functional foods, has suggested that they are difficult to categorise for both regulators and users. It is argued that they blur the boundary between ‘food' and ‘drug' and that uncertainties about the products create ‘experimental' or ‘restless' approaches to consumption. We investigate these uncertainties drawing on data about the use of functional foods containing phytosterols, which are licensed for sale in the EU for people wishing to reduce their cholesterol. We start from an interest in the products as material objects and their incorporation into everyday practices. We consider the scripts encoded in the physical form of the products through their regulation, production and packaging and find that these scripts shape but do not determine their use. The domestication of phytosterols involves bundling the products together with other objects (pills, supplements, foodstuffs). Considering their incorporation into different systems of objects offers new understandings of the products as foods or drugs. In their accounts of their practices, consumers appear to be relatively untroubled by uncertainties about the character of the products. We conclude that attending to materials and practices offers a productive way to open up and interrogate the idea of categorical uncertainties surrounding new food products.

## Introduction

A wide range of foods are now marketed with specific health claims, and appear to both regulators and social scientists to sit somewhat uneasily on the border between ‘food' and ‘medicine'. While some analysts have suggested that they render this border meaningless, leading to the medicalisation of eating, others have examined the conceptual work done by regulators and consumers to maintain it, or manage categorical uncertainties. Scholarship on novel foods suggests three sets of criteria for distinguishing between food and drugs: the intended purpose of a product; its intrinsic properties; and its mode of use. While the first two of these have featured in scholarly discussion, we think there is scope for further consideration of the third – how products are used in practice. This article considers how the formulation and packaging of a specific set of functional foods promising cholesterol reduction is regulated, and how the resulting products are domesticated through their incorporation into everyday routines. It explores what purchase can be gained on understanding categorical uncertainties from the perspective of materiality and material practices.

In this article we draw on an analysis of regulatory and marketing texts, and of the packaging of the products as well as 40 in-depth interviews with current or former users of products containing plant sterols living in the United Kingdom. We suggest that the regulation and marketing of the products embedded a set of expectations and instructions about foods containing plant sterols, scripting these as medicinal to be used in a targeted and dosed fashion, but this scripting was largely at odds with the ways they were domesticated.

Users' accounts suggested that rather than approaching the products as distinct objects for a specific purpose and in a calculative way, they were incorporated into everyday life as part of a system of objects (for example, margarine might be tied to the consumption of bread, where the frequency of consumption might be guided by a number of considerations). In this way, phytosterols were domesticated in ways that made sense within everyday practices rather than the scripts incorporated in the products and packaging. However scripts for some products were stronger than for others. For example, the small size of yogurt drinks and the composition of the product scripted a once per day ‘dose' that most interviewees could not see as contributing to eating or drinking. The identity of these products as supplements rather than food or drink was reinforced in accounts where they were bundled into everyday pill/supplement-taking routines. While interviewees might categorise products containing plant sterols in terms of qualities such as natural or unnatural, safe or harmful, tasty or not so palatable, looking at the material form of the products and considering users' accounts of their everyday practices opens up an alternative way of understanding the meaning of these products for consumers.

## What are Functional Foods and What Kinds of Boundaries do they Disrupt?

Functional foods, a term first coined in Japan in the 1980s, have no standard definition. Though the term may be employed in relation to unprocessed foods, in Europe it has come to be used for products that are marketed on the basis of health-promoting claims that go beyond nutritional value and that have been produced through a process of research and development (Heasman and Mellentin, 2001; Niva, 2008). Products containing phytosterols are a prominent example of this category, marketed as foods that lower blood cholesterol and therefore promote heart health. Phytosterols, which are plant derivatives, have been incorporated into a range of foods including, for example, margarine spreads, yogurt drinks, yogurts and cheeses.

Analysts have argued that functional foods have disrupted both established regulatory and lay understandings of food and drugs. For example, Landecker (2011, p. 185) suggested that through the consumption of foods like functional foods and nutrigenomics “the distinction between food and drug is becoming blurred and with it the distinction between eating and medicating”. Scrinis (2008) offers a more overtly critical voice arguing that these products contribute to a pathologised, reductionist and medicalised approach to food. Similarly Holm (2003) suggests that the idea of functional foods rests on the notion of individual and technical rationality, that is, that properly informed consumers will apply appropriate nutritional science to their daily life to maximise their health. As others have commented, this requires consumers to adopt a reflexive and calculative approach to eating (Beardsworth and Keil, 1997; Debevec and Tivadar, 2006; Niva, 2008). Yet, as Holm (2003) argues this means that other rationalities aligned with eating – practical, economic, symbolic and relational – are ignored.

These debates have also been rehearsed during the development of new regimes of regulation for functional foods, which have confronted numerous categorical uncertainties. Until the 1990s commercial products were generally classified on the basis of their intended use (or purpose) so that any products marketed with claims to treat or prevent disease or affect physiological processes were likely to be classed as medicines (Termini, 1993; Prothro, 1997). Lehenkari (2003, p. 500) argues that Benecol margarine, the first phytosterol product to be launched “came outside established categories and challenged them, thus creating debate and even conflicts during national approval processes”. Recognising the increasing difficulty of maintaining a distinction between food and medicine, food regulation has created new categories. In the United States the category of ‘dietary supplements' was introduced (Dietary Supplement Health and Education Act, 1994), and these do not require Food and Drug Administration approval. Measures have been introduced in both the United States and EU to sanction specifically worded health claims for other products (Nutrition Labeling and Education Act, 1990; Regulations (EC), 258/1997, 608/2004, 1924/2006) on the basis of standards of evidence reminiscent of those required for drug licensing (Regulation (EC), 353/2008; Hasler, 2008). In the case of phytosterols this evidence has proven the ability to lower cholesterol (under trial conditions) although not cardiovascular disease (European Food Safety Authority, 2008a, 2008b).

In contrast to the regulatory focus on intended use or the purpose of the products, research with ‘consumers' or users suggests that they may categorise products according to certain intrinsic qualities, which are already associated with the categories of food or medicine. Thus Britten (2008) summarises, ‘natural' remedies tend to be seen as both safer and more gentle than pharmaceuticals, a point reinforced by Nichter and Thompson (2006) who report that dietary supplements may be seen as more natural and safer than pharmaceuticals, with fewer side effects. These qualities also appear in research specifically on people's responses to phytosterols, which suggests that they are not easy to categorise by their intrinsic qualities. Drawing on survey and focus group data from Finland, Niva (2008) suggests that her respondents saw phytosterol products as sitting uneasily between the categories of ‘natural' and ‘technological' and even ‘healthy' and ‘unhealthy' because of their processed nature. Further focus group research in Britain, Denmark and Sweden underlines participants' difficulties in understanding functional foods, which challenge their distinctions between healthy/unhealthy and natural/unnatural, and occupy an “anomalous” (Korzen-Bohr and Jensen, 2006, p. 152) or “ambiguous” (Landström *et al*, 2009, p. 40) position between food and medicine – for Jauho and Niva (2013) functional foods are best understood as ‘hybrids' of the two. Where Nichter and Thompson (2006) suggested that consumers responded to uncertainties around dietary supplements by “experimenting”, Lezaun and Schneider (2012, p. 370) draw on other (market) research to argue that European consumption of functional foods is characterised by a kind of “restlessness” as the “standard” of naturalness or healthfulness shifts with the proliferation of choices.

## Approaching the Use of Functional Foods

While studies focused on the meanings or understandings of functional foods suggest categorical uncertainties matter to potential consumers, as Niva (2008) comments this does not preclude their use. There are a number of studies reporting on who uses functional foods and why (that is, *intended purpose from a consumer perspective*). Niva's (2007) work on Finnish consumers finds that foods containing phytosterols are mostly used because of an identified cholesterol problem, thus suggesting a highly targeted and medicine-like approach. In a review paper, Jauho and Niva (2013) discuss additional questions regarding how a substance is used. They suggest that in lay evaluations medicines are seen as being used in exact doses and draw attention to the advertising of some functional foods that are presented like “a course of treatment with medicines, which also stresses regularity and the correct dosage” (ibid., p. 52). We infer that this may offer a further criterion by which the categorical uncertainties described above may be resolved. Thus the distinction between food and drugs depends not only on their *qualities* and *intended purpose*, but also on *modes of use*. Evidence from surveys however suggests that many users of phytosterols do not have high cholesterol, that this varies by country, and that people often eat less than recommended for effectiveness (Simojoki *et al*, 2004; TNS, 2006; de Jong *et al*, 2007).

In our own interviews with UK phytosterol users (Weiner, 2011), in addition to responding to specific prompts such as cholesterol test results or reacting to a perceived family history of heart disease, we identify a number of other rationales for buying or eating these products including general health consciousness, a sense of doing something good for oneself or habit. This recalls Holm's (2003) observation about the coexistence of multiple rationalities shaping ordinary eating practices, and suggests less targeted and (technically) rational purposes than those rehearsed by Niva's (2007) Finnish respondents. Resonating with Holm (2003), we have observed sustained multiplicity in people's accounts of their everyday eating practices (Will and Weiner, 2014) and have also argued that health practices (which include the use of supplements and functional foods) may be described as a form of ‘patchwork' or ‘do-it-yourself', a practical rather than rational endeavour built up over time (Will and Weiner, 2013).

To summarise the literature then, critical analysts of functional foods have suggested that functional foods promote a medicalised view of eating and imply that they have already changed the way people approach food, blurring the boundary between food and medicine. Categorical uncertainties about functional foods have appeared to matter both in regulation and in research on (potential) consumers' responses to such products which have often focused on the ‘qualities' attributed to these foods. At the same time, findings relating to consumers' intended purposes of use have varied, but suggest the possibility of less (technically) rational and targeted motivations than implied by the idea of medicine. We think there are still questions about the ways in which functional foods are used as part of everyday practices. In order to gain further purchase on the idea of functional foods as uncertain products in this article we consider in more detail what people do with phytosterols in a very mundane and everyday sense. This involves thinking about the material form of the products and the practicalities of everyday life. In the next section we introduce some theoretical resources for this project, before detailing our methodology and then our findings.

## Attending to Materials and Practices

Theoretically our analysis is situated in Science and Technology Studies (STS) but draws on different strands of this tradition than either Landecker's (2011) account of scientific discourse and practice, or Lezaun and Schneider's (2012) analysis of the regulatory work of qualification. Instead, we wish to draw attention to the material presence of functional foods as objects that are picked from supermarket shelves, brought into people's homes and workplaces, and incorporated into everyday practices.

We start from the way in which the material form of an object or product is designed to convey particular messages, a process described by Akrich (1992) as a matter of “scripting” the preferred identity of potential users and their uses of a technology. These scripts are embedded in the very “technical content” (ibid., p. 208) of an object. Here, textual instructions for use are only part of the script that may also include the shape, size and material nature of constituent components (Latour, 1991, 1992; Akrich, 1992). These material characteristics may suggest, constrain or impel particular actions in stronger or weaker ways. This approach has gained wide currency within STS. However in STS objects are only ‘durable' to the extent that new sites reproduce previous patterns of relations or practices – materiality is ‘relational' (Law, 2008). Thus the original discussion of ‘scripts' also sought to leave space for users to de-scribe these prescriptions through what are called ‘anti-programmes' or attempts at ‘re-inscription' (Latour, 1991; Akrich, 1992; Akrich and Latour, 1992). This has variously been described with the relatively conflictual language of resistance (see Kline and Pinch, 1996), the more comfortable sounding ‘domestication' (Silverstone *et al*, 1992; Lie and Sørensen, 1996; Carter *et al*, 2013) or celebratory references to user creativity and improvisation (Bruni *et al*, 2013).

Recent theoretical work has also urged that the ‘script' metaphor needs to be used carefully. Darr and Pinch (2013, p. 1612) argue for a “less restrictive notion of material script” than proposed by Akrich, so that rather than design, STS would focus on ‘material scripts' as “a recurrent pattern of interaction involving material objects in a specific setting”. Here script seems to include people's own embodied sense of what should be done with an object. This resonates with other work calling for the analysis of ‘technologies in practice' (Timmermans and Berg, 2003). The idea of ‘practices' has gained considerable momentum in STS work that focuses on material objects in everyday life. A good example is work by Shove *et al* (2007) on the ways in which domestic technologies (for example, kitchen appliances, digital photography) are incorporated into social practices. They provide a useful summary of how STS can explore the importance of materiality in practice, arguing that “we need to pay … attention to the ways in which artefacts relate to each other and to the part humans and non-humans play in configuring variously stable material taxonomies and variously durable systems of objects” (Shove *et al*, 2007, p. 11). It is this claim that is the jumping-off point for our analysis.

## Our Study

Studying the use of functional foods presents methodological challenges since a large part of this takes place in private, domestic spaces and over a long time frame. The relevant actions are therefore not readily amenable to observation (Bryman, 2001; Murphy and Dingwall, 2003). Like Nichter and Thompson we used semi-structured interviews to talk with people who identified themselves as currently or formerly buying or eating these foods. Weiner undertook the interviews as part of an ongoing collaboration between the authors on everyday practices of cholesterol management.

We recruited on a pragmatic basis largely through a university and an older people's network in two different cities in England. We advertised the study to a variety of academic and non-academic staff groups, including manual and technical staff, by e-mail and poster and via the quarterly newsletter of the older people's network. Weiner undertook 40 interviews with a total of 45 current or former consumers, and sometimes with other members of their household, between 2009 and 2010. Participants had a range of occupational backgrounds and ages, though, as would be anticipated (TNS, 2006), most were over 40. Our informants were established users of a variety of products, largely from ranges by Benecol or Flora pro.activ, which are the two major UK brands. Most bought or ate spreads (38), but about a third used other products in addition (drinks, yogurts, milk or cheese).

Interviews were undertaken in people's homes (just over half), or in their own or Weiner's workplace. Interviews undertaken in participants' own settings allowed for a material inflection to conversations on occasions, for example when interviewees fetched products from their refrigerator, produced and talked through their collection of supplements, or opened an office refrigerator to show the storage of yogurts.

Interviews were fairly loosely structured, following a topic guide that Weiner employed flexibly. Overall, this was intended to elicit talk about how people came to use the products, how they were incorporated into daily routines, wider practices around eating and health, health biographies and interactions with health-care professionals. Interviews opened by asking participants how they came to buy or eat the foods, which prompted accounts of use and non-use over time. Talk about how people incorporate the foods in their daily life was encouraged using a mixture of general questions, for example, tell me about when you eat them and why then?, and specific questions, for example, can you tell me what you ate yesterday – take me through the day?; tell me about the last occasion you ate any [phytosterol]? This approach was intended to elicit detailed accounts of practices. Because of an awareness of critiques of functional foods in the literature, Weiner sometimes asked an explicit question towards the end of the interview about whether the participants saw the products as food or drug in order to encourage reflection on this issue.

In these interviews we were interested in both people's descriptions of their everyday practices as well as their reflections on these, although of course in people's talk these are mixed together. We recognise the limits of interviews for researching practice (Hammersley and Atkinson, 1995; Dingwall, 1997) but believe that here they have provided important insights into the mundane and material aspects of everyday actions involving plant sterols. We have discussed elsewhere how the use of these products fitted into participants' wider everyday health practices (Will and Weiner, 2013, 2014).

In addition to carrying out and analysing interview data, we compiled documents for a critical understanding of the debates surrounding the products' emergence and regulation (see Weiner, 2010 for details of method). We also gathered material to allow analysis of the packaging and marketing of the different products. Weiner audited the products available at her local supermarket and purchased examples of different types to scrutinise the packaging. We gathered print advertisements from 1999 onwards from three general interest magazines that regularly advertise the products, available from the British Library's collection (Gardener World, BBC Good Food Magazine and Radio Times) and also studied the UK Websites of Flora pro.activ and Benecol.^1^ We draw across these data to explore the categorical uncertainties claimed to surround functional foods along with other novel food products from the perspective of users.

The article looks first at ‘scripts' in the sense that Akrich proposes, looking at the instructions embedded in the material form of phytosterols, first in elements prescribed by regulation, and second in the marketing of specific products. The third, fourth and fifth sections consider how the products were actually used, drawing on a broader concept of material scripts as developed by Pinch and others, as well as the concepts of domestication, improvisation and practice. We show that users were unlikely to take a calculative approach to use, and rarely saw themselves as attempting to consume a measured dose. We then consider the way the products were incorporated into domestic routines by being linked with other objects, for example, margarine was spread on bread, or drinks were consumed together with pills, and these systems of objects helped define the products. Finally we explore how, when asked explicitly about the categories to which the phytosterols belonged, people reasoned in a relational way, making use of distinctions between food and medicine to situate the products. In all of this, participants drew upon their own sense of ‘material scripts' for a wider range of household goods, as well as the characteristics of the phytosterols themselves.

## Regulation: Interventions in Material Form

EU regulations concerning functional foods include a number of practical stipulations about how they may be presented to the consumer in the United Kingdom (Regulations (EC) 258/1997, 608/2004, 1924/2006). Manufacturers have sought permission under these regulations to market a range of different types of products, incorporating phytosterols into spreads, milks, yogurts, yogurt drinks and cheese. In giving permission for specific health claims to be made about these products, regulators set out quite detailed instructions about the size of packaging and written information to be included alongside any images or branding. Here we will focus on the ‘scripts' carried by packaging across the ranges.

According to the EU framework, packaging of all these products must include written statements, which might reduce the uncertainty felt by consumers about the value and use of phytosterol products. They must state that the product is intended exclusively for people who want to lower their blood cholesterol, provide a definition of a portion or serving size of the food and amount of the plant sterol that each portion contains, and advise that the consumption of more than 3 g/day of added phytosterols should be avoided.^2^ Regulation concerning functional foods therefore attempts to reduce uncertainty about the use of the products by providing explicit written ‘scripts' on the packaging. In the case of phytosterols this ties them relatively firmly to a specific health concern (with raised blood cholesterol) and offers written instructions about a maximum recommended daily intake. As Jauho and Niva (2013) note, this focus on dose as well as insistence that use should be motivated by a specific health concern does position the products as medicine-like.

However, though these scripts are explicit, and carefully regulated, we suggest here that they are relatively ‘weak', for nothing compels consumers to actually read them, let alone act on the instructions. Regulators seem to acknowledge some of the limitations of such written advice in relation to the issue of appropriate intake through making further stipulations about their packaging. Where possible, foods containing phytosterols should be provided in single portions, containing either the maximum daily recommended amount or one-third of it, or should give a clear indication of the size of a ‘standard portion'. These requirements support the idea that there is an appropriate ‘dose' for the foods to be effective in the stated aim of lowering cholesterol.

Studies produced in evidence for this regulatory approval typically tried to manage the uncertainties around the effective amount and what was consumed by providing their participants with plant sterol products packaged in individual portions for daily consumption (for example, 8 g packs of margarine, 3 to be consumed per day; 25 g packs of margarine, 1 to be eaten per day) (European Food Safety Authority, 2008a, 2008b). Thus, in these trials, dose was scripted through the provision of portioned packages.

## Marketing: Making Phytosterol Products

When putting phytosterols onto the market, manufacturers formulated them in a range of different types of ‘food', with different kinds of packaging. Yogurt drinks or dairy-free drinks were presented in small single portions as desired by the regulators (100 g or fewer) containing the whole of the daily recommended amount of phytosterols, and packs of individual pots of yogurts (around 100 g) each provided a third of the daily recommended amount. However they sold other products in much larger packets. Indeed the most popular product in our sample (and in market research (TNS, 2006)) was spreads, which were sold in 250 and 500 g packets – here 10–12 g constitutes a ‘serving' (that is, one-third of recommended daily amount). Similarly, milk was sold in litre packets (where 250 ml constitutes a ‘serving') and cheese in packets of 195 g (where a ‘daily serving'=65 g).

As required, written nutritional information and guidance about ‘portions' asked consumers to take a calculative approach to eating, and be sure to take enough but not too much (like a medicine). However the material form of the products was also important for the eventual script. The packaging of drinks, in particular, left little uncertainty or space for calculation, by creating single ‘doses'. We might say that the drinks are relatively ‘strongly' scripted, like a pill. By contrast, spreads have a much ‘weaker' script. Written advice is given, both in small text on the underside of the packets (see Box 1) and brief information about ‘portion size' on the lid (see Box 2). Yet such written information seemed to be less compelling than the strong scripts of the package size.

The scripting of the different products was supported to some extent by their wider marketing. Advertising campaigns in general have focused on the cholesterol-reducing properties of the products. Initial marketing of Benecol in the United Kingdom in 1999 introduced the idea of a ‘cholesterol-reducing diet' (adding Benecol) in contrast to a ‘reduced cholesterol diet' (no spread/butter). Later campaigns of both Benecol and Flora pro.activ have focused on ‘real people' and their success stories – focusing more on the cholesterol reductions achieved rather than on the specific products and their uses. Brand Websites^1^ both currently feature the idea that cholesterol can be reduced by up to 10 per cent in 3 weeks by incorporating their products, offering this as a challenge and providing ‘help' to people to achieve this, including wider lifestyle information. Nevertheless, print adverts for yogurt drinks/dairy-free drinks sometimes, although not always, included the idea of a single daily portion in more or less prominent ways. For example, an advert launching the new Benecol yogurt drink in 2004 prominently features the words “once a day” under a picture of the bottle, and the text “new Benecol yogurt drink, proven to reduce cholesterol”. Other marketing of drink products by both brands have focused on their specific qualities such as “dairy free” or “probiotic and cholesterol lowering” while including the idea of one bottle per day within a block of smaller text. Yet an advert in 2007 for a Flora pro.activ drink does not include any text about portion size, focusing only on cholesterol lowering.

## From Intended Use to Practices: The Limits of Calculation

We now turn to our data about what people actually did with the products once they had been taken off supermarket shelves and brought into the home. Following the logic of the regulation, and Jauho and Niva's (2013) suggestion of one basis for lay distinctions, we investigated whether people were calculative in their use, that is, whether they were attempting to consume the optimum recommended dose.

The only case where people talked about being aware of actually consuming the recommended dose was in discussing the drinks, where people sometimes recognised the labelling edict of *one-a-day*:

Interviewer: Do you have them everyday or what do you do? INT36: Yes, it tells you to have one a day, mm.

Yet those who had the drinks, even some who recognised the one a day script, might still consume other phytosterol products as well, suggesting that the dosage script held only for the specific product.

We found there was rarely a sense that people might eat more or less of other products on a given day in order to consume the recommended amount. The written and relatively ‘weak' script about appropriate intake was hardly ever referred to, and the marketing idea of ‘servings' and of consuming two to three servings of spread, yogurt or milk per day was absent. We can illustrate this using the narrative of the middle-aged woman who was using phytosterols as part of a specific project to manage her cholesterol levels:

INT2: I have the milk on cereal for breakfast, and then all my, not at work, but all my other drinks at home I have, but I don't drink it on its own, I don't like Interviewer: And didn't you say you eat the margarine? How often do you have that? INT2: I might just have a piece of toast a day with that spread on it, or if I have a sandwich or something, but then I might not have a sandwich everyday or if I have, like, new potatoes.

Though this woman hoped to reduce her cholesterol with the products, there was no sense that she kept track of the amounts of the different products she consumes or that she tracked the cumulative consumption across the products, seeking to take a particular ‘dose' at one meal or across the day as one might for a medicine. Her use was framed by other logics (being at home or work, if having toast or a sandwich) and by the tastes that she enjoyed or sought out on a particular day. It was difficult to sustain a sense of something that you spread on bread as medicine, not least because of the different ways in which bread was consumed over a day or week.

While people could talk about the frequency with which and occasions when they ate the products, there was hardly any quantification talk and this idea clashed with strongly embedded eating practices. One rare example of a spread user who had read the instructions on the package commented, you ‘can't force yourself' to eat more of the margarine:

INT33: Well I read about the recommended amounts are per day 25 grams […]. But of course I can't force myself to eat a lot, you know more than 25 a day, I eat my bread in a morning with some spread and bring my sandwich with the spread […]

Although the products may be initiated for purposes that align with scientific and regulatory guidance, such as cholesterol reduction, they were rarely used in the targeted and calculative way this guidance assumed.

## Situating Phytosterols in Systems of Objects

As the data on margarine use in the previous section illustrate, using functional foods often meant incorporating them into existing practices. We describe these practices in this section in the terms of Shove *et al* (2007) as a matter of bringing functional foods into more or less “durable systems of objects” in everyday life.

In the case of spreads, ‘use' depended largely on people's consumption of bread, though it was occasionally eaten with potatoes or in other cooked food. Bread consumption might fluctuate over the week or other timeframes depending on work patterns or its durability (see Will and Weiner, 2014). Talk about the amount of spread consumed most often came up in relation to efforts to reduce bread consumption (with the knock on effect that less spread was needed) or reduce how much fat was eaten. Jauho and Niva (2013) comment that fat is usually regarded as something to be avoided and it may be difficult for people to see it as cholesterol reducing. Here the cholesterol-lowering properties are less in question, rather eating practices are linked to other, perhaps competing, aims such as weight management or reducing gluten consumption. So, for example, one woman described having spread only at the weekends because she did not eat bread in the week “because it helps me keep my weight down”.

People were adept at improvising with other foods to make a sandwich appropriately ‘moist' while limiting fat intake:

INT19W: If you're having a sandwich you don't always need to put something on the bread if the filling is moist you know you don't necessarily need to put fat on it. So that reduces the amount of fat you eat. INT21: Well the spread is very, very infrequently because one of the things that I try to do is not have spread or butter or anything, if I'm having a sandwich I usually have something like perhaps some mustard or some chutney or something like that on my bread just to flavour it a little bit, together with the filling.

It was important that the phytosterols in spread could not be consumed without other foods, drawing them into systems of objects and practices of food preparation, for example, no spread if filling is moist, or conventions about eating at different times or places, for example, whether having a sandwich or cooked meal, eating more healthily in the week, having more time for breakfast at the weekends. Here, the use of phytosterols and the weak scripts accompanying the milk and margarine in particular, came up against other more or less material scripts, rationales and conventions, about appropriate meals, palatable sandwiches and weight reduction.

This was not the case for drinks. These products were still incorporated into everyday routines, but they were rarely consumed as part of a meal, and were seen as extra to normal eating or serving little nutritional purpose. In some cases they were incorporated into other systems of objects, together with pills or supplements:

INT39: I have a drink every morning yeah it's just part of a routine now […] normally I'm going down to make the sandwiches, […] and then my good lady normally comes down and she lays out my tablets and my drink […] I just drink it because I see it as, I take that tablet, that tablet, I drink that juice and I drink that […] Well normally I have the little drink, I have my vitamin pills and I have a drink of cranberry juice to clear my mouth, that's it, me done.

Thus the phytosterol drinks may be enacted as supplements or medicines through their positioning in particular practices – and even ‘cranberry juice' enlisted in instrumental action (clearing mouth). In another example of this positioning of drinks as part of pill-taking practices the distinction between Benecol and food was underlined through one woman's reflection that she does not eat in the mornings – making clear that for her Benecol does not contribute to eating.

INT1: Well I try, in the mornings, the plan is to have a Benecol and take my beta blocker every morning, one bottle, and I don't eat in the morning.

This feature was important for users in further conceptual talk in response to an explicit question about whether the phytosterols were ‘food' or ‘medicine' – and the categories were developed through talk about practices of shopping, meal planning or pill-taking, and the objects incorporated in their practices, rather than phytosterols' qualities.

INT30: I don't really see it as part of my diet, that's very true […] I think of it as a medicine I think. Interviewer: Right so are you able to articulate what makes it a medicine? INT30: I suppose it's that as I'm thinking of my meals throughout the day and planning my shopping and planning my menu a bottle of Benecol would not be one of the things that I'd have on the list, does that make sense, do you know what I mean? So it is, it's a bit like the multivitamin pill and mum's Eye Wise [supplement] that I swallow, it's sitting with those.

If the drinks were not part of ‘planning my menu' they were more medicine-like, or perhaps supplement-like (it ‘sits with vitamins'). In the following example, the same logic is used to make an even clearer distinction between the cheese and spread as foods ‘something to be used normally in everyday life', in contrast to yogurt drinks that are perceived as medicines:

INT34(husband): I've always thought that about the little drinks, it's almost like they're trying to sell you medicine, so I do perceive them as being medicine […] for me personally yes the cheese and the spread are something I would normally use in everyday life I consider food and yes perhaps if I ate yogurt I might consider the yogurts to be food. But yeah these drinks and anything of that nature […] INT34(wife): I do perceive them as a medicine as opposed to, I do like yogurt but I wouldn't have it in drink form, normally I'd rather have it with a spoon with some fruit in and perhaps over some fruit, as a pudding […]. INT34(husband): But you wouldn't think as part of my meal I'll have a little bottle of something would you?

However, people could create new routines that did link the drink with other objects or foodstuffs – practices that might be described as forms of ‘improvisation'. For example, one woman talked of pouring the yogurt drink onto her porridge, in the same way that one might pour on a dash of cold milk:

INT8: I thought it was quite nice so I just had it on my porridge with soya milk every morning.

Here the product was found a role in this interviewee's breakfast routine. More generally, however, it seemed difficult for people to incorporate the little drinks into ‘meals' and therefore to define them as foods. On the other hand, where spreads were used as part of sandwiches, in baking or with vegetables, milk poured into tea or onto cereal and yogurts eaten for pudding, they appeared to be accepted as food. In both cases the products acquired their significance as part of larger systems of other objects consumed as part of domestic routines.

## Reflecting on Categories: The Use of Material Taxonomies

In this final findings section we draw on further data that came in response to an explicit question about whether phytosterol products are food or medicine. Echoing previous research, this sometimes produced talk about the qualities associated with the products. It is striking however that these were not limited to the claims about the naturalness or safety, but also to the experience of some products as more pleasurable than others.

For some users of the spreads finding pleasure in the taste helped make the product at least partly a food.

INT12: I try to enjoy food as well, it's still nice what we have […]. Interviewer: Is that a food or a medicine, how do you see it? INT12: I'd definitely say both. I mean it tastes fine and when you put a bit extra on I enjoy it, it's not going to harm me, it might do us a bit of good.

When people found the spread ‘perfectly palatable' but ‘not delicious to eat', it might be seen more like a medicine:

Interviewer: Do you think about the Flora pro.activ as a food or as a medicine or is it something in between or is it not possible to answer that question? INT21: I suppose in between. Yes I wouldn't take it if I didn't think it was going to do some good, I'd just stick with butter. I mean it's perfectly palatable but I prefer the taste of butter to Flora pro.activ, so I suppose I veer more on the side of medicine rather than a, something that's delicious to eat.

Again the drinks were somewhat easier for most users to categorise. In this quote it was implied that there was no enjoyment in the drink and it certainly could not count as pudding or dessert:

Interviewer: How do you see it, do you see it as like a INT16: A medicine [laughs] No I see it like a medicine, I just swig it down. Interviewer: Okay so it's not, it's not like a, your pudding or your sweet? INT16: A dessert, no I usually have fruit for my pudding.

When people were pushed explicitly to discuss the boundary between food and medicine they drew on their experiences of pleasure in the taste. Products might be seen as more medicine-like because they were thought not to taste very nice or conversely as less medicine-like when they were experienced as tasting good.

Nevertheless others did define particular products through more conceptual qualities such as naturalness or artificiality, in particular when drawing contrasts with medicines. For example, some people talked about choosing to use phytosterols to reduce cholesterol in preference to pills. In the example below a couple (husband and wife) talk about the wife's consumption of plant sterol margarine:

INT26(husband): [wife] is using it as an alternative to having tablets, she'd rather try something of the food type rather than the pure medical type […] you're worried about side effects. INT26(wife): Well I think it's a better way to live, to do things naturally than to be on medication […]

In contrast, occasionally people expressed discomfort about the phytosterols as being ‘processed' or ‘artificial' in comparison with butter:

INT17: I've always only used natural products, like sort of you know butter rather than spreads because I think they were less processed and less artificial […] I do keep thinking about it because I actually don't like taking pro.activ, it feels like I'm taking medication.

## Discussion and Conclusion

We started this article with the categorical uncertainties surrounding functional foods highlighted by analysts, regulators and (potential) lay users. We also identified the different ways of distinguishing between foods and drugs drawn on in discussions of these uncertainties: intended use or purpose, intrinsic qualities and mode of use. In our analysis we have sought to draw attention to the material form of the products, in terms of their packaging and presentation, as well as the ways they were incorporated into everyday practices and located in systems of objects, in order to open up a different perspective on questions about shifting boundaries between foods and medicines.

Previous work has examined regulation as a site for making conceptual distinctions and raised questions about whether functional foods sit between food and medicine, or cross the boundary between them (Lehenkari, 2003; Landecker, 2011). In our analysis we drew attention to the ways in which this regulation seeks to make these concepts material through requiring particular information to be included in labels on products containing phytosterols and giving instructions about the packaging size and formulation.

We suggested that while both regulation and product marketing could be understood as attempts to provide ‘scripts' for consumption, such scripts were stronger for some products than for others, especially when the package related directly to the written instructions about serving size. To put this in Akrich's (1992) terms, they suggested a different division of responsibility between the object and the user, where some products take responsibility for users imbibing the recommended amount of phytosterols and other products appearing to offer more latitude, or leave this responsibility to the human consumer.

Drawing on our interviews with users of different phytosterol products in the United Kingdom, we were then able to present data on the “material scripts” described by Darr and Pinch (2013) or what Nichter and Thompson (2006) call “use in context”. These included material scripts for using functional foods, but also other everyday items including sandwiches, pills and cups of tea. In these sections we drew attention to features of practice that seemed to move some phytosterol products away from the category of medicine and towards food, for some people. A key issue here was calculation: our respondents made only limited reference to the idea of getting an appropriate dose (cf. Jauho and Niva, 2013). The weak scripts about appropriate servings and daily consumption that we identified in both labels and elements of the packaging and formulation of spreads, yogurts and milk rarely featured in these interviews. The packaging and formulation of the small drinks might be read as a stronger script for a daily dose. Often, however, the ‘scripts' incorporated in product packaging were overlooked in favour of existing and overlapping practices associated with food preparation, eating and weight management.

In our view, consumers' diversion from the intended use of these products is better characterised as ‘domestication' than ‘resistance', because, like ‘de-scribing' or ‘anti-programmes', resistance suggests a degree of conscious opposition to producers' scripts. Domestication starts with the world of the user (Oudshoorn and Pinch, 2003), and draws attention to the material as well as conceptual dimension of use, as people described how products had been incorporated into their domestic routines. Like Carter *et al* (2013), we found that people had to work to accommodate new objects into daily routines, and suggest that, here, this involved bundling the product into what Shove *et al* (2007) call “systems of objects”, which included other foods, drinks and pills or supplements. Spreads and other products like milk, cheese and yogurt were consumed as part of meals or snacks (bundled with other foodstuffs), following conventions about appropriate daily eating practices. In this sense they were treated as foods and not as medicines. The individual bottles containing yogurt drinks made these products hard to incorporate into the preparation of a meal. Indeed their small size meant they generally did not appear satisfying as a drink or even snack. On occasions, the identity of these drinks emerged quite vividly through descriptions of their incorporation into existing routines for taking pills or supplements, thus placing them with pills and supplements in a material as well as conceptual sense. More occasionally, people described improvising to find places for them by combining with foods, such as pouring a yogurt drink onto porridge, though this was not the skilled and intentional deviation from a script that is signalled in the discussion of improvisation in Bruni *et al* (2013).

Attending to the way the different products were domesticated leads us to suggest that the drinks were relatively strongly scripted as supplements/medicines, not only because of their dosage into once per day portions, but because of their small size that seemed largely to preclude their incorporation into eating practices. Further, we venture that margarine offers not just a weak but also a contradictory script from a user perspective not only because the base food is high in fat (cf. Jauho and Niva, 2013) but because of the way margarine is incorporated into systems of objects and practices, where cholesterol management is but one focus, and because package size does not align clearly with written instructions.

Our discussion of the material form of phytosterol products, and the ways in which they were combined into different systems of objects, thus adds specificity to discussions about functional foods. Though research has focused on functional foods in total or particular classes of these products (as we have in this study), consumers may treat one product containing phytosterols quite differently from another, indicating that this is not a distinct ‘class' from a consumer perspective. Individual phytosterol products may belong to different categories when viewed from the perspective of practice, as well as the ‘scripts' embedded in the formulation and presentation of the product by regulators and manufacturers. However this point comes with important qualifications. Though regulators paid most attention to the specific writing containing health claims and instructions for use, which was to be incorporated into packaging, other decisions about the material form, especially the size of the packaging appeared to act as ‘stronger' scripts for eventual use, though these competed with existing eating practices and expectations.

We suggest then that attending to practices gives quite a different impression of the consumption of phytosterols than might have been implied by regulation or market research (cf. Lezaun and Schneider, 2012) or by the health claims made for new functional foods (Lehenkari, 2003; Landecker, 2011). While marketing and regulation appeared to present them as boundary-crossing products, our informants, who were established users, narrated the relatively unproblematic incorporation of products into existing practices of eating or medicating. Embedding a product in set of material relations (for example, for pill-taking or sandwich-making) created more or less stable routines for the domestic consumption of specific functional foods. Though people could produce talk about the ‘qualities' of particular products, especially in relation to their naturalness (as predicted by Niva, 2008 and Lezaun and Schneider, 2012), they appeared relatively untroubled by what Jauho and Niva (2013) suggest is the ‘hybrid' quality of the phytosterols. Indeed strong contrasts between food and medicine could be used as a resource: for those who disliked pills, phytosterol products were presented as a ‘non-medical' alternative; those who disliked processed food saw them as medicines. Importantly, relevant qualities also included the taste of particular products, meaning people drew on their embodied experience and their pleasure in eating (see also Koteyko, 2010). When people liked the taste, functional foods were less likely to be seen as medicines than products that people found did not taste as good as butter. As highlighted in other work in STS on eating, pleasure can then remain an important attribute of food even when eating practices are inflected with health concerns (Vogel and Mol, 2014).

To conclude, unlike Landecker (2011) we argue that the distinction between eating and medicating was still relatively clear for our respondents, though phytosterols might be consumed in ways that made them either food or medicine, depending on the household and on the particular product. We end then with the suggestion that research on functional foods, and other foods, should attend to the complexities of practice as well as regulation and consumers' understandings in more abstract terms. Though participants in this study could rehearse uncertain boundaries, when they told us how they used different products – their practices – these fell away. Attending to what people do with functional foods and their reasoning tied to concrete instances of practice allowed us to suggest different kinds of distinctions and offer alternative ways of thinking about categorical uncertainties said to accompany the introduction of functional foods.

## Example of scripting: very small text on underside of Flora pro-activ margarine tub.

Enjoy Flora as part of a varied and balanced diet including lots of fruit and vegetables, and a healthy lifestyle and your heart can benefit. Each 10 g serving of Flora pro-activ spread contains 0.75 g of plant sterols. Health experts agree that 2–2.5 g of plant sterols a day is optimal for cholesterol lowering. More than 3 g is not recommended.

## Footnotes

1 For more details of the product ranges and examples of the marketing of the products see www.benecol.co.uk/ and www.floraproactiv.co.uk/proactiv/

2 Weiner (2010) suggests that stipulations concerning maximum recommended dose and targeting on people with high cholesterol related as much if not more to a set of moral and social concerns about potential users' health-related behaviours, than to concerns about safety or toxicity.
